# The EWAS Catalog: a database of epigenome-wide association studies

**DOI:** 10.12688/wellcomeopenres.17598.1

**Published:** 2022-02-04

**Authors:** Thomas Battram, Paul Yousefi, Gemma Crawford, Claire Prince, Mahsa Sheikhali Babaei, Gemma Sharp, Charlie Hatcher, María Jesús Vega-Salas, Sahar Khodabakhsh, Oliver Whitehurst, Ryan Langdon, Luke Mahoney, Hannah R. Elliott, Giulia Mancano, Matthew A. Lee, Sarah H. Watkins, Abigail C. Lay, Gibran Hemani, Tom R. Gaunt, Caroline L. Relton, James R. Staley, Matthew Suderman

**Affiliations:** 1 MRC Integrative Epidemiology Unit, University of Bristol, Bristol, BS8 1TH, UK; 2 Bristol Medical School, University of Bristol, Bristol, BS8 1TH, UK; 3 Centre for Exercise, Nutrition and Health Sciences, University of Bristol, Bristol, BS8 1TH, UK; 4 Bristol Renal, Translational Health Sciences, Bristol Medical School, University of Bristol, Bristol, UK

**Keywords:** EWAS, epigenome-wide, epigenetics, database, ALSPAC

## Abstract

Epigenome-wide association studies (EWAS) seek to quantify associations between traits/exposures and DNA methylation measured at thousands or millions of CpG sites across the genome. In recent years, the increase in availability of DNA methylation measures in population-based cohorts and case-control studies has resulted in a dramatic expansion of the number of EWAS being performed and published. To make this rich source of results more accessible, we have manually curated a database of CpG-trait associations (with p<1x10 ^-4^) from published EWAS, each assaying over 100,000 CpGs in at least 100 individuals. From January 7, 2022, The EWAS Catalog contained 1,737,746 associations from 2,686 EWAS. This includes 1,345,398 associations from 342 peer-reviewed publications. In addition, it also contains summary statistics for 392,348 associations from 427 EWAS, performed on data from the Avon Longitudinal Study of Parents and Children (ALSPAC) and the Gene Expression Omnibus (GEO). The database is accompanied by a web-based tool and R package, giving researchers the opportunity to query EWAS associations quickly and easily, and gain insight into the molecular underpinnings of disease as well as the impact of traits and exposures on the DNA methylome.

The EWAS Catalog is available at http://www.ewascatalog.org.

## Introduction

EWAS assess associations between traits of interest and DNA methylation across the genome ^1– 3^. These associations may be used to gain mechanistic insights into disease and developmental processes or serve as molecular biomarkers in prediction applications ^1– 3^. Giving researchers easy access to the data will likely improve understanding of complex traits and may yield other translational benefits.

The EWAS Atlas has previously collated well-curated EWAS on traits in an online database and makes annotated CpG site-level results accessible via a website ^4^. Other databases are available but are limited to certain diseases (e.g. MethHC ^5^).

Ideally, a database of EWAS results will provide summary statistics, including effect estimates, standard errors, and p-values in an easily accessible manner, so that researchers can explore associations without having to retrieve the original article. For example, allowing comparison of effect estimates between studies or a look-up of specific associations to evaluate replication. For completeness, such a database should also, where possible, provide summary statistics for all potentially true associations beyond those passing conservative significance thresholds, but publications rarely report sub-threshold lists of associations. The contents of EWAS Atlas have to-date been restricted to published associations.

**Figure 1.**
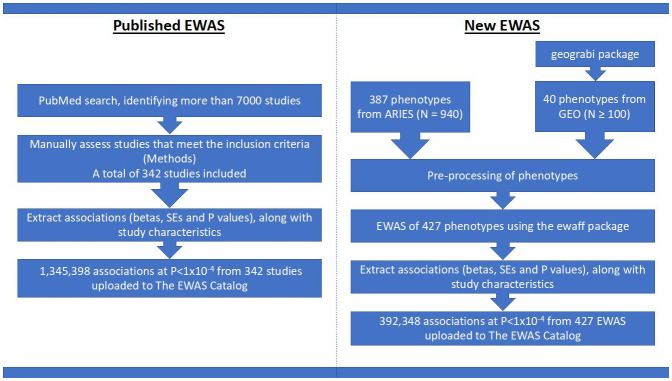
Project flowchart. On the left is a brief description of how we assembled CpG-phenotype associations from published works and on the right is a brief description of the EWAS performed using individual level data.

We therefore aimed to improve upon current databases to 1) provide all relevant summary statistics from a range of EWAS and 2) allow easy and programmatic access to results. To this end we have produced The EWAS Catalog, a manually curated database of currently published EWAS with additional data from 387 EWAS performed in ALSPAC ^6, 7^, and 40 EWAS performed with publicly available data from the GEO database. The process and data inclusion are summarized in Figure 1. The EWAS Catalog also enables users to upload results, which go through manual and automated checks ensuring the data meets the standards of the database, allowing collection of results not necessarily reported in publications.

## Methods

### Implementation

The EWAS Catalog web app was built using the Django Python package ( https://djangoproject.com). The data is stored in a combination of a MySQL database and fast random access files ^8^, and can be queried via the website or the R package.

### Overview of publication data extraction

To identify publications, we perform periodic literature searches in PubMed using the terms: "epigenome-wide" OR "epigenome wide" OR "EWAS" OR "genome-wide AND methylation" OR "genome wide AND methylation".

Our criteria for inclusion of EWAS are as follows:

1. The EWAS was performed using data from over 100 humans.
2. The analysis contains over 100,000 CpG sites
3. DNA methylation data is genome-wide (not a candidate gene study)
4. Results are not duplicated from a previous study
5. CpG-trait associations at p<1x10 ^-4^ are reported

These criteria and the variables extracted are documented on the website. Briefly, extracted variables included: the exposure variable, the outcome variable, the covariates, tissue, sample size, age, sex, reported ancestry or ethnicity, CpG IDs, effect estimates, standard errors, p-values. To unify representation of traits, they were mapped to Experimental Factor Ontology (EFO) terms, which were manually extracted from the European Bioinformatics Institute database.

### EWAS study data

***GEO datasets Avon Longitudinal Study of Parents and Children (ALSPAC)*.** EWAS were conducted for 387 continuous and binary traits ( *Extended data* ^9^) using DNA methylation measured in peripheral blood of middle-aged ALSPAC mothers (N = 940). The trait data were extracted from information collected at the same sampling point blood was drawn for DNA methylation assays. Quality control steps for the traits and information on the cohort are in the Extended methods section.

***GEO datasets*.** Full EWAS results were also estimated for studies that did not report complete summary statistics in their initial publication but where complete DNA methylation and trait of interest information were publicly available through the GEO database. We used the geograbi R package to query GEO for experiments matching inclusion criteria (described above) and extract data for EWAS re-analysis. The query was performed using the geograbi.retrieve.datasets() function on 12 October 2020 and identified 136 experiments with 32,555 samples meeting The EWAS Catalog inclusion criteria where DNA methylation and phenotype information could be successfully extracted. GEO identifies publications corresponding to all database records by PubMed ID and we accessed these for all retained GEO datasets to identify the original variable of interest. We aimed to replicate the original published analysis from the available GEO data in order to generate a full set of summary statistics to be included in The EWAS catalog. However, of our 136 putative GEO studies, only 34 (25%) contained sufficient information to replicate the original analysis. The main reason for study exclusion at this stage was for missing phenotype information. Both original published results and the full re-analysed GEO results have been included in The EWAS Catalog database. A list of all 40 traits with corresponding citations is provided as *Underlying data* ^10^.

Details on the statistical analyses for EWAS performed specifically for The EWAS Catalog can be found in the Extended methods section. The full summary statistics for these results can be found on the following Zenodo projects: https://doi.org/10.5281/zenodo.4672645, https://doi.org/10.5281/zenodo.4672754.

As of January 7, 2022, The EWAS Catalog contained 1,737,746 associations from 2,686 EWAS.

### Extended methods

***Avon Longitudinal Study of Parents and Children (ALSPAC)*.** Pregnant women residing in Avon, UK with expected dates of delivery 1st April 1991 to 31 ^st^ December 1992 were invited to take part in the study. The initial number of pregnancies enrolled was 14,541 (for these at least one questionnaire has been returned or a “Children in Focus” clinic had been attended by 19 July 1999). Of these initial pregnancies, there was a total of 14,676 foetuses, resulting in 14,062 live births and 13,988 children who were alive at 1 year of age. Full details of the cohort have been published previously ^6, 7^. The EWAS performed for The EWAS Catalog were done so using DNA methylation measured in peripheral blood of ALSPAC mothers in middle age (N = 940), generated as part of the Accessible Resource for Integrated Epigenomics Studies (ARIES) project ^11^.

All continuous and binary phenotypes were extracted from the same timepoint that blood was drawn for DNA methylation assays. A list of the phenotypes can be found in the *Extended data* ^9^.

Ethical approval for the study was obtained from the ALSPAC Ethics and Law Committee and the Local Research Ethics Committees. Consent for biological samples has been collected in accordance with the Human Tissue Act (2004). Informed consent for the use of data collected via questionnaires and clinics was obtained from participants following the recommendations of the ALSPAC Ethics and Law Committee at the time. The study website contains details of all the data that are available through a fully searchable data dictionary and variable search tool.

***Preparing phenotype data from ALSPAC and GEO for EWAS*.** For continuous traits we defined outliers as follows:


Outlier<LQ+3∗IQROutlier>UQ+3∗IQR,

where LQ = lower quartile, IQR = interquartile range, UQ = upper quartile. Any outliers were set to missing, then all phenotypes with 100 or more non-missing values were kept for further analysis. To ensure all phenotypes were approximately normally distributed, each distribution was examined and transformed as required. Log-transformations were performed on right-skewed variables. Square-roots and cube-roots were used to try and approximate normality if log-transformation did not produce an approximately normal distribution. To produce approximately normally distributed data for left-skewed variables, they were squared.

***EWAS statistical analyses*.** For all EWAS performed specifically for the EWAS Catalog, linear regression models were fit with DNA methylation as the outcome, coded as numbers between 0 and 1, and the trait as the exposure. For EWAS using ARIES participant data, covariates included age, the top 10 ancestry principal components, and 20 surrogate variables (SVs). For EWAS using GEO data, 20 SVs were included as covariates. Other covariates were considered, but SVs only were used for two reasons: 1) to help automate the process and 2) because covariates used in the original EWAS were not included with many GEO datasets.

Analyses were conducted in R (Version 3.6.2). The smartsva package ^12^ was used to create SVs and the ewaff R package was used to conduct the EWAS; all p-values are two-sided.

## Results

**Figure 2.**
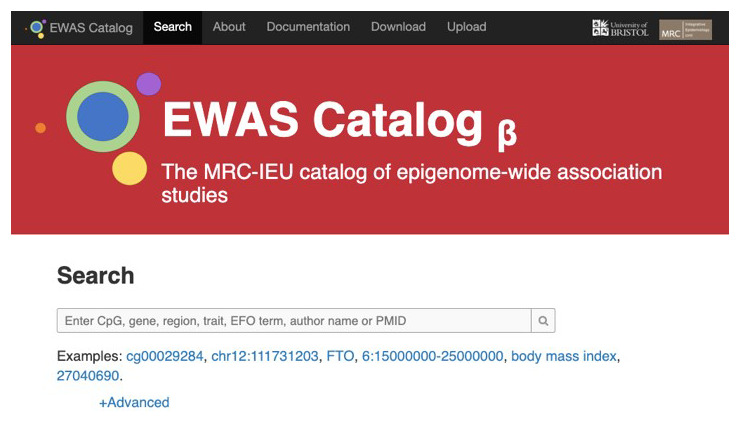
The EWAS Catalog home page. From here users can search the database, view documentation and navigate to pages that allow for download of the full database and upload of user results. An example of results can be found in Figure 3.

**Figure 3.**
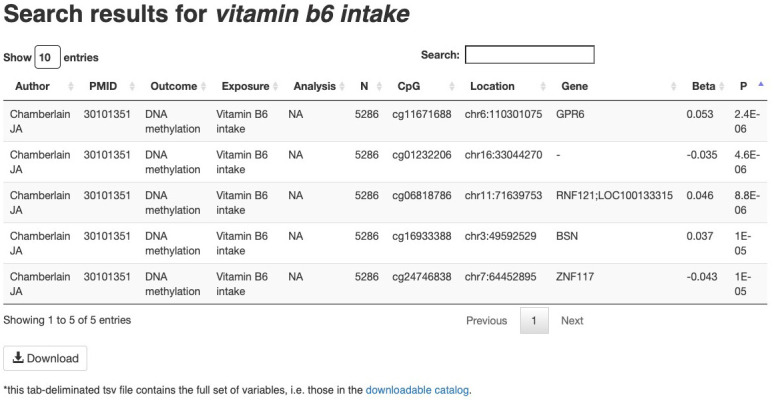
Example of results from The EWAS Catalog website. These results can be extracted by clicking the “Download” button at the bottom of the figure. This download will include extra study information, such as age, sex and reported ancestry of study participants.

The database can be queried at www.ewascatalog.org. The website provides a simple user interface with a search bar to explore the database as well as documentation on the catalog contents and how to cite its use ( Figure 2). Basic queries may include a CpG identifier, gene symbol, genome region, trait, author name or PubMed ID. Query submission will then lead to an intermediate ‘splash page’ providing options for more specific queries. For example, a query for a specific trait would lead to a ‘splash page’ listing that trait, related traits, and all studies of that trait. Selecting one of these leads to a list of relevant EWAS associations, including CpG ID, trait, sample size, publication, and association (effect size and p-value) ( Figure 3). This information, along with further details such as reported ancestry, outcome, exposure units and tissue analyzed, are available for download as a tab-delimited text file. Alternatively, advanced queries are also supported wherein both a CpG identifier, gene symbol or genomic region are specified along with a trait, author name or PubMed ID. These queries are more specific and lead directly to a list of relevant EWAS associations.

The catalog can also be queried programmatically using the “ewascatalog” R package. Installation instructions and examples are available at its Github repository. Once installed, the database can be queried directly in R using the “ewascatalog()” function similarly to the website. By supplying the function with a CpG site, gene, genome position or trait, the function returns the same output as is downloadable from the website.

## Discussion/conclusions

The EWAS Catalog provides a database of summary statistics from currently published EWAS and an additional 427 currently unpublished EWAS. This database has similar aims to the EWAS Atlas but has additional data sources, provides extra useful information and a user upload option. The EWAS Catalog team will continue to collate and upload newly published EWAS and perform additional EWAS on available datasets, whilst encouraging EWAS authors to upload their own summary data. We are currently working to incorporate additional functionality to allow users to systematically compare their own EWAS findings to EWAS already in the database.

## Data availability

### Underlying data

The EWAS Catalog URL: http://www.ewascatalog.org

All published summary statistics at p<1×10 ^-4^ are available on the website. Any additional statistics or data associated with publications can be obtained by following links to the publications provided by The EWAS Catalog website. The full summary statistics from all EWAS conducted within ALSPAC, GEO and from any uploaded data can be found here: https://zenodo.org/communities/ewas-catalog. The original GEO data can be found on the GEO website ( https://www.ncbi.nlm.nih.gov/geo/) using the accession IDs provided as underlying data ( https://doi.org/10.5281/zenodo.5905938) ^10^ and The EWAS Catalog website or R package.

ALSPAC data is accessed through a system of managed open access.

The steps below highlight how to apply for access to the data included in this software tool article and all other ALSPAC data. The data presented in this article are linked to ALSPAC project number B3259, please quote this project number during your application. The ALSPAC variable codes highlighted in the dataset descriptions can be used to specify required variables.

1. Please read the ALSPAC access policy (PDF, 627kB) which describes the process of accessing the data and samples in detail, and outlines the costs associated with doing so.
2. You may also find it useful to browse our fully searchable research proposals database, which lists all research projects that have been approved since April 2011.
3. Please submit your research proposal for consideration by the ALSPAC Executive Committee. You will receive a response within 10 working days to advise you whether your proposal has been approved.

If you have any questions about accessing data, please email alspac-data@bristol.ac.uk.

The ALSPAC data management plan describes in detail the policy regarding data sharing, which is through a system of managed open access.

The project contains the following underlying data:

Zenodo: The EWAS Catalog manuscript: Underlying data https://doi.org/10.5281/zenodo.5905938 ^10^

### Extended data

This project contains the following extended data:

- A table of the 387 traits for which EWAS were conducted using data from ARIES along with the sample sizes for each of the EWAS: The EWAS Catalog manuscript: Extended data ( https://doi.org/10.5281/zenodo.5905767) ^9^

Data are available under the terms of the Creative Commons Attribution 4.0 International license (CC-BY 4.0).

## Software availability

Source code available from: https://github.com/MRCIEU/ewascatalog

R package available from: https://github.com/MRCIEU/ewascatalog-r

Archived R package code as at time of publication: https://doi.org/10.5281/zenodo.5519348

License: MIT
