# Supplementary material for: The EWAS Catalog: a database of epigenome-wide association studies
Source: Wellcome Open Res. 2022 May 31;7:41. Originally published 2022 Feb 4. [Version 2] doi: 10.12688/wellcomeopenres.17598.2 (PMC9096146; doi:10.12688/wellcomeopenres.17598.2)
Supplement: Peer review history

# Peer review: Reviewer response for version 1

**DOI:** 10.21956/wellcomeopenres.19460.r50168

**Published:** 2022-05-11

**Authors:** Harold Snieder

**Affiliations:** 1 Department of Epidemiology, University Medical Center Groningen, Groningen, The Netherlands

This is a clear software tool article introducing the EWAS catalog. I have a few questions and suggestions the authors may want to consider.

1. The 5th inclusion criterion specifies that CpG trait associations at p<1x10 ^-4^ need to have been reported in order for the study results to be included in the EWAS catalog. What is the justification for this criterion (make it more explicit) and is it wise to apply it strictly? I’m afraid interesting studies may be missed that have not reported CpG trait associations at p<1x10 ^-4^.
2. It should be made more attractive for researchers to upload full EWAS summary statistics to the EWAS catalog, so it becomes the go-to repository for scientists that like to build on earlier work through making use of available EWAS summary stats and perform meta-analyses, just like the GWAS catalog is for GWA studies. As such, please mention that it is one of the aims of the EWAS catalog to encourage scientists to upload their EWAS summary stats, make the upload procedure as user friendly as possible and provide more information on this procedure in the current article.
3. The catalog is periodically updated with new publications, but with which frequency? Every month?
4. Were all the GEO EWAS conducted in peripheral blood or also other tissues?
5. For the new EWAS analyses in ALSPAC and GEO, why was DNA methylation specified as the outcome rather than the reverse. Please indicate briefly pros and cons.
6. Are the 20 surrogate variables (SVs) expected to sufficiently capture technical covariates such as batch effects and cell type distribution? A supporting reference would help.

Are the conclusions about the tool and its performance adequately supported by the findings presented in the article?

Yes

Is the rationale for developing the new software tool clearly explained?

Yes

Is the description of the software tool technically sound?

Yes

Are sufficient details of the code, methods and analysis (if applicable) provided to allow replication of the software development and its use by others?

Yes

Is sufficient information provided to allow interpretation of the expected output datasets and any results generated using the tool?

Yes

Reviewer Expertise:

(Epi)Genetic epidemiology

I confirm that I have read this submission and believe that I have an appropriate level of expertise to confirm that it is of an acceptable scientific standard.

# Response

**Published:** 2022-05-26

**Authors:** Thomas Battram

**Affiliations:** University of Bristol, UK

*This is a clear software tool article introducing the EWAS catalog. I have a few questions and suggestions the authors may want to consider.*

 We thank the reviewer for their positive comments, and helpful suggestions and questions. See our responses to each one individually below.

- *The 5th inclusion criterion specifies that CpG trait associations at p<1x10-4 need to have been reported in order for the study results to be included in the EWAS catalog. What is the justification for this criterion (make it more explicit) and is it wise to apply it strictly? I’m afraid interesting studies may be missed that have not reported CpG trait associations at p<1x10-4.*

It is true that CpG-trait associations at P>1x10 ^-4^ may be of interest. Ideally, we would store full-summary statistics that could be searched. Unfortunately, we don’t have the capacity to store such a large dataset. Hence, when inputting newly published EWAS into our database, we contact the authors and offer the service of uploading full summary statistics to Zenodo when uploading their data to The EWAS Catalog. We have added a link to these data in the “Download” section of the website to improve their visibility. For studies that report results in their paper at P>1x10 ^-4^, we do not ask for the results to be uploaded to Zenodo as the data can be accessed via the original paper. We provide PubMed IDs that make it easy to link EWAS to their publications. Further, we have found that it is rare that individuals report EWAS associations at P>1x10 ^-4^, thus the majority of data in The EWAS Catalog database is all that could be taken from each study. Hopefully this will change in the future and people will report full summary statistics. We plan on continuing to upload these full summary statistics to Zenodo.

 The P<1x10 ^-4^ threshold is somewhat arbitrary, but it does have some justification. It corresponds to 80% power to detect a 10% difference in DNA methylation in a sample set of size of 100 (50 per group) assuming a standard deviation of 0.1 for DNA methylation. Given that this ignores adjustment for multiple tests (typical EWAS perform hundreds of thousands of tests) and that 10% methylation differences are rare to observe in an EWAS, we consider this a very permissive threshold.

- *It should be made more attractive for researchers to upload full EWAS summary statistics to the EWAS catalog, so it becomes the go-to repository for scientists that like to build on earlier work through making use of available EWAS summary stats and perform meta-analyses, just like the GWAS catalog is for GWA studies. As such, please mention that it is one of the aims of the EWAS catalog to encourage scientists to upload their EWAS summary stats, make the upload procedure as user friendly as possible and provide more information on this procedure in the current article.*

We thank the reviewer for the great suggestion. Currently, we try to encourage users to upload EWAS summary statistics by contacting the authors of new EWAS every month and asking if they would upload. We’ve attempted to make the process as simple as possible by providing a short online form to complete and an upload link for summary statistics. To increase visibility of the platform and to remind others that they can upload summary statistics, we’ve now made a Twitter profile for The EWAS Catalog ( https://twitter.com/ewascatalog). And will shortly begin regularly tweeting about catalog updates and relevant highlights from the literature. We’ve also added these lines to the end of the abstract:

 “The EWAS Catalog data extraction team continue to update the database monthly and we encourage any EWAS authors to upload their summary statistics to our website. Details of how to upload data can be found here: http://www.ewascatalog.org/upload.”

 And we have added these lines to the end of the results:

 “To upload data to The EWAS Catalog database, authors need to simply fill out a short form describing the study (inputting the meta-data variables listed in the Overview of publication data extraction section) and then upload the EWAS summary statistics (effect estimates, standard errors, and P values). The link to this online form and the location of where to upload the summary statistics can be obtained by emailing “ewascatalog@outlook.com”, and one of our team will promptly respond with all the necessary details.”

- *The catalog is periodically updated with new publications, but with which frequency? Every month?*

The catalog is updated monthly. This information has now been added to the website, ( http://www.ewascatalog.org/about/). We’ve further incorporated a line on the front page of the website that indicates when the data was added. We also added this line to the end of the “Overview of publication data extraction” section:

 “The EWAS Catalog data extraction team extracts data from newly published EWAS monthly.”

- *Were all the GEO EWAS conducted in peripheral blood or also other tissues?*

Great question. The GEO EWAS were conducted in multiple tissues. We have added the lines below the “EWAS study data” section to indicate the tissues used. This information can also be seen when downloading the data from The EWAS Catalog database.

 “Half of the 40 EWAS, measured DNA methylation data in whole blood, and there was a range of tissues used for the other EWAS, including saliva, brain, skin, colon. This data is available as part of the downloadable meta-data from The EWAS Catalog website.”

- *For the new EWAS analyses in ALSPAC and GEO, why was DNA methylation specified as the outcome rather than the reverse. Please indicate briefly pros and cons.*

This modelling decision was entirely practical. Due to the large number of EWAS conducted, we did not want to hypothesise a direction of effect for each trait. However, evidence from previous studies suggests effects are more likely to go from trait to DNA methylation rather than the other way around (Wahl et al. Nature, 2017; Min et al. Nature Genetics, 2021).

 For the EWAS run using the data from ALSPAC, blood was drawn to measure DNA methylation at the same time as phenotypes were measured. Therefore, knowing whether a change in DNA methylation occurred before trait variation is difficult. For the GEO data, it was unclear from the majority datasets when the DNA methylation was measured.

 From a statistical point of view, the set of top associations identified by an EWAS should be identical whether or not DNA methylation is the outcome. Having DNA methylation as the outcome is also convenient as it ensures that the outcome is always continuous. Of course, effect sizes will differ in magnitude and interpretation, but we consider this less important than identifying top CpG site associations.

- *Are the 20 surrogate variables (SVs) expected to sufficiently capture technical covariates such as batch effects and cell type distribution? A supporting reference would help.*

Surrogate variables will capture the factors in the data that contribute most to DNA methylation variation independent of the trait of interest. Previous studies have shown that batch effects are major components of DNA methylation variation, effects that surrogate variable analysis was first designed to identify. Cell proportions have also been shown to contribute greatly to DNA methylation variability and surrogate variables have been shown to partially capture this source of variability as well. We’ve now added the following to the manuscript at the end of the “Extended methods” section:

 “SVs were included in our EWAS models to capture unmeasured confounded factors, especially batch effects and cell composition differences. SVs were originally developed to help identify batch effects (Leek and Storey, Plos Genetics, 2007) and are commonly used in EWAS to do this (Teschendorff and Relton, Nature Reviews Genetics, 2018), but they’ve also been shown to capture cell composition differences (Teschendorff and Relton Nature Reviews Genetics, 2018; Kong et al. Plos One, 2019).”

# Peer review: Reviewer response for version 1

**DOI:** 10.21956/wellcomeopenres.19460.r49012

**Published:** 2022-03-14

**Authors:** John W. Holloway

**Affiliations:** 1 Human Development and Health, Faculty of Medicine, University of Southampton, Southampton, UK; 2 NIHR Southampton Biomedical Research Centre, University Hospital Southampton, Southampton, UK

Battram *et al.* describe the construction of an on-line database, the “EWAS catalogue”, that provides a tool to allow researchers to query EWAS associations quickly and easily. The database is populated with data from 342 published studies along with data generated *de novo* from the ARIES epigenetics dataset of middle-aged women in ALSAPC (N=367 phenotypes) and EWAS data generated from geo datasets (N=40).

 The documentation is clear and the website well presented. Importantly, an option is provided for authors to upload their own datasets, allowing for further enrichment of the database going forward.

 The database presented is complementary to the EWAS atlas (Li M *et al*. 2019), especially given the inclusion of the ALSPAC and Geo data.

 Have the authors considered providing an API interface to allow integration of the EWAS catalogue into other tools? What about a trait enrichment tool so researchers can check for enrichment of a set of CpGs identified (e.g. associated with an exposure) with disease associated CpGs in the catalogue?

Are the conclusions about the tool and its performance adequately supported by the findings presented in the article?

Yes

Is the rationale for developing the new software tool clearly explained?

Yes

Is the description of the software tool technically sound?

Yes

Are sufficient details of the code, methods and analysis (if applicable) provided to allow replication of the software development and its use by others?

Yes

Is sufficient information provided to allow interpretation of the expected output datasets and any results generated using the tool?

Yes

Reviewer Expertise:

Genetic and Epigenetic epidemiology

I confirm that I have read this submission and believe that I have an appropriate level of expertise to confirm that it is of an acceptable scientific standard.

# Response

**Published:** 2022-05-26

**Authors:** Thomas Battram

**Affiliations:** University of Bristol, UK

*Battram et al. describe the construction of an on-line database, the “EWAS catalogue”, that provides a tool to allow researchers to query EWAS associations quickly and easily. The database is populated with data from 342 published studies along with data generated de novo from the ARIES epigenetics dataset of middle-aged women in ALSAPC (N=367 phenotypes) and EWAS data generated from geo datasets (N=40).*

*The documentation is clear and the website well presented. Importantly, an option is provided for authors to upload their own datasets, allowing for further enrichment of the database going forward.*

*The database presented is complementary to the EWAS atlas (Li M et al. 2019), especially given the inclusion of the ALSPAC and Geo data.*

 We thank the reviewer for their positive comments and excellent suggestions. See our responses below.

- *Have the authors considered providing an API interface to allow integration of the EWAS catalogue into other tools?*

Yes, we currently have an API to access The EWAS Catalog from R. It is still experimental so we haven't advertised it yet. It can be installed from Github: https://github.com/MRCIEU/ewascatalog-r.  

- *What about a trait enrichment tool so researchers can check for enrichment of a set of CpGs identified (e.g. associated with an exposure) with disease associated CpGs in the catalogue?*

This is an excellent idea. We have begun work on implementing this tool and have a version that works offline. We are preparing a manuscript describing results obtained using that tool. We hope to provide website access to the tool soon.
